# Editorial: Molecular host-pathogen interactions in veterinary infectious diseases: from pathogenesis to immunomodulatory therapies

**DOI:** 10.3389/fvets.2026.1972998

**Published:** 2026-09-09

**Authors:** Lei Na, Mo Zhou, Tong-Yun Wang

**Affiliations:** 1 College of Animal Husbandry and Veterinary Medicine, Jiangsu Vocational College of Agriculture and Forestry, Jurong, China; 2 School of Biopharmaceuticals, Jiangsu Agri-Animal Husbandry Vocational College, Taizhou, China; 3 Department of Cellular and Molecular Medicine, University of California (UC) San Diego School of Medicine, La Jolla, CA, United States

**Keywords:** host immunity, host-pathogen interaction, immunomodulatory therapy, novel vaccines, pathogen immune evasion, veterinary infectious diseases

Veterinary infectious diseases impose massive economic losses to global livestock, aquaculture and companion animal industries, while continuously raising zoonotic spillover risks to human populations (1). Conventional disease control strategies centered on antibiotics and universal vaccination face growing limitations: widespread antimicrobial resistance, incomplete cross-protection of traditional vaccines, and emerging/re-emerging pathogens with evolved immune escape mechanisms collectively demand paradigm shifts in disease intervention (2). Deciphering molecular host-pathogen crosstalk represents the core breakthrough point to resolve these bottlenecks, as it uncovers how pathogens manipulate host transcriptional programs, cell fate and immune signaling, and guides the design of host-targeted, immunomodulatory interventions beyond pathogen-eliminating agents (3). This Research Topic assembles high-quality original research spanning viral, bacterial pathogens of swine, ruminants and aquatic animals, systematically dissecting infection mechanisms and translating basic molecular discoveries into practical anti-infection strategies, while strictly excluding epidemiological surveys and pure diagnostic assays as defined by our scope criteria.

A central theme across collected works is pathogen-mediated transcriptional reprogramming and tissue tropism regulation at the molecular level. One study focused on exogenous Jaagsiekte sheep retrovirus (exJSRV), the causative agent of ovine pulmonary adenocarcinoma, mapped the core transcriptional regulatory motif (−146/−132) within viral LTR and identified GATA3 as the dominant host transcription factor activating LTR transcription, with MEK/ERK signaling acting as a positive upstream modulator (Du et al.). *In vivo* bioluminescence imaging further verified exJSRV LTR preferential tropism toward mouse lung and liver tissues, explaining the pulmonary tumorigenic preference of exJSRV driven by tissue-enriched GATA3/FoxA transcription factor pools. This work clarifies a conserved host transcription factor-viral cis-element axis underlying retroviral oncogenesis, offering novel molecular targets for blocking ovine lung tumor progression.

Another representative transcriptomic investigation systematically characterized staged regulated cell death (RCD) cascades during PRRSV-2 infection in MARC-145 cells via time-series RNA-seq (Cao et al.). PRRSV sequentially orchestrates distinct cell death modalities: early inflammatory priming coupled with suppression of lytic cell death (necroptosis, ferroptosis), mid-phase ER stress-triggered apoptosis and flux-impaired autophagy, and late-onset cuproptosis/alkaliptosis driven by metabolic dysregulation. Persistent immunogenic cell death signatures were sustained throughout infection, revealing a delicate viral strategy to balance self-replication and delayed host cytolysis. This temporal RCD atlas provides phase-specific therapeutic windows for immunomodulatory anti-PRRSV interventions targeting metal ion homeostasis and ER stress pathways.

Complementing viral pathogenesis studies, a comparative genomic and immunization work explored host-driven divergence of *Trueperella pyogenes* pyolysin (PLO) virulence factors isolated from deer and swine strains (Zhu et al.). The swine-origin strain carries unique accessory virulence loci and harbors 19 amino acid substitutions in PLO protein, altering surface B-cell epitopes and structural stability. Mouse immunization trials demonstrated deer-derived recombinant PLO elicited faster early humoral immune responses and superior post-challenge survival, whereas swine PLO induced stronger pro-inflammatory cytokine overshoot with limited protective efficacy, highlighting that strain-specific PLO epitope variations shape differential protective immunogenicity. This finding strongly advocates conserved epitope screening for broad-spectrum multi-antigen anti-*T. pyogenes* subunit vaccines.

Beyond pathogen intrinsic virulence mechanisms, this Research Topic also delivers translational functional research for eco-friendly immunoregulatory feed additives against aquatic bacterial diseases. A probiotic fermented herbal preparation using *Bacillus amyloliquefaciens* was optimized via single-factor fermentation parameter screening; in largemouth bass feeding and *Nocardia seriolae* challenge trials, the composite additive significantly elevated growth performance, serum antioxidant enzyme activity, systemic immune gene expression and intestinal microbial diversity, while achieving 77% relative survival post-infection and complete clearance of granulomatous lesions in vital organs (Li et al.). It establishes a sustainable antibiotic substitute for aquaculture disease prevention via dual immunostimulation and gut microecology remodeling.

Collectively, all included manuscripts converge on the core logic: dissecting molecular host-pathogen interactions enables rational design of immunomodulatory therapeutics, replacing outdated pathogen-centric control modes. Current contributions lay solid groundwork, yet multiple critical gaps remain unaddressed, including parasitic/fungal pathogen crosstalk, primary host cell models (e.g., porcine alveolar macrophages) verification of cell line-derived discoveries, and large-animal translational validation of candidate vaccines/therapeutics. We anticipate follow-up research expanding this Research Topic's coverage, further bridging molecular pathogenesis and field disease prevention, and supporting One Health-oriented control of veterinary infectious diseases.
